# Sirtuins in Women’s Health

**DOI:** 10.3390/ph18121859

**Published:** 2025-12-05

**Authors:** Rasajna Madhusudhana, Abu Hamza, Emily Boyle, Shannon Pollock, Yana Cen

**Affiliations:** 1 Department of Medicinal Chemistry, Virginia Commonwealth University, Richmond, VA 23219, USA; madhusudhanr@vcu.edu (R.M.); hamzaa@vcu.edu (A.H.); boyleel2@vcu.edu (E.B.); pollocksg@vcu.edu (S.P.); 2 Center for Drug Discovery, Virginia Commonwealth University, Richmond, VA 23219, USA

**Keywords:** sirtuin, women’s health, metabolic, aging, estrogen

## Abstract

The human sirtuins (SIRT1–SIRT7) are NAD^+^-dependent protein deacylases that orchestrate key cellular events such as metabolism, stress response, DNA repair, and aging. Accumulating evidence highlights their central role in women’s health. This review integrates recent insights into the roles of sirtuins across the female lifespan and their involvement in reproductive, metabolic, oncologic, and age-related disorders. Sirtuins regulate reproductive function, pregnancy outcomes, and hormone-dependent cancers. Their decline with aging contributes to menopausal and metabolic complications. Pharmacological interventions that enhance sirtuin activity, such as NAD^+^ precursors and SIRT1 activators, show promise in mitigating these conditions. Collectively, understanding the isoform- and tissue-specific roles of sirtuins provides a foundation for developing therapeutics to improve the lifespan and healthspan of women.

## 1. Introduction

The seven human sirtuins (SIRT1–SIRT7), a family of NAD^+^-dependent protein deacylases, have emerged as critical regulators of cellular homeostasis, metabolism, inflammation, and aging [1]. Their diverse biological functions encompass DNA repair, mitochondrial biogenesis, oxidative stress response, and epigenetic regulation [2], mechanisms that are intricately linked to numerous health conditions. Recent research increasingly underscores the relevance of sirtuins in sex-specific physiology and pathology, particularly in the context of women’s health.

Women experience a unique spectrum of health challenges across their lifespan, influenced by hormonal fluctuations, reproductive cycles, and aging processes [3,4]. These include reproductive disorders such as polycystic ovary syndrome (PCOS), endometriosis, and infertility; pregnancy complications such as preeclampsia and gestational diabetes; and postpartum conditions including depression. Furthermore, breast and gynecologic cancers, including ovarian, cervical, and uterine cancers, represent a significant global health burden. Later life stages introduce new risks associated with menopause and osteoporosis.

Emerging evidence positions sirtuins as the key modulators in the pathogenesis and progression of these female-specific conditions. Their ability to regulate inflammation, insulin sensitivity, oxidative stress, mitochondrial biogenesis, and hormonal signaling highlights their potential as promising therapeutic targets. However, the complexity of their functions and the interplay between different sirtuin isoforms demand a careful understanding of their context-dependent effects.

This review provides an overview of the multifaceted roles of sirtuins in women’s health. We integrate insights from molecular biology, preclinical studies, and emerging clinical data to examine the involvement of sirtuins across various gynecologic, metabolic, oncologic, and age-related disorders. Special attention is given to research published during the last five years. Our aim is to highlight their translational potential and identify opportunities for future research and therapeutic development.

## 2. Polycystic Ovarian Syndrome (PCOS)

PCOS is a prevalent endocrine disorder affecting approximately 6–13% of women of reproductive age [5]. It is a leading cause of infertility and is associated with various pregnancy complications [6]. The hallmark clinical features of PCOS include anovulation, irregular menstrual cycles, enlarged ovaries with multiple peripheral cysts, hyperandrogenism, and insulin resistance (IR) [7]. In addition, affected individuals often experience weight gain, hirsutism, dermatological issues, and mood disorders [8,9,10]. The etiology of PCOS is multifactorial, involving a complex interplay of genetic, epigenetic, environmental, and lifestyle factors [11]. Systemic inflammation and oxidative stress also play critical roles in its pathogenesis [12]. PCOS is a common comorbidity in women with obesity, diabetes, and other metabolic disorders, highlighting its strong metabolic component [13].

Combined oral contraceptives and aromatase inhibitors are commonly used to manage hyperandrogenism, while insulin sensitizers are employed to address IR [14]. However, many current pharmacological treatments for PCOS are associated with adverse effects, limited efficacy, poor adherence over the long term, and frequent contraindications [15].

Sirtuins, key regulators of inflammation, oxidative stress, and steroidogenesis, have emerged as potential targets for PCOS treatment [16]. SIRT1 and SIRT2 are downregulated in granulosa cells of PCOS models [17,18], while mitochondrial sirtuins show variable expression [19]. These alterations are linked to metabolic dysfunction, disrupted folliculogenesis, and altered reproductive morphology [20,21,22]. Recent studies focus on sirtuin modulators and their downstream effectors in PCOS.

### 2.1. SIRT1

SIRT1 activates key regulatory proteins such as forkhead box protein O1 (FOXO1), pyruvate dehydrogenase kinase 4 (PDK4), and serine/threonine kinase 11 (LKB1) while also suppressing pro-inflammatory cytokines like nuclear factor kappa B (NF-κB) through deacetylation and AMPK-dependent signaling pathways [17]. These actions collectively support proper follicular development, glucose metabolism, and the regulation of oxidative stress and inflammation. In PCOS, androgen excess leads to SIRT1 downregulation, disrupting the AMPK–SIRT1 axis [17,23,24]. Pharmacological modulation of this axis, using natural compounds and GLP-1 receptor agonists, has shown promise in treating metabolic syndrome [25], prompting interest in targeting SIRT1/AMPK signaling to manage multiple aspects of PCOS.

Several natural compounds including scoparone, catalpol, fisetin, sulforaphane, quercetin, and resveratrol (Table 1) have been studied in letrozole- or androgen-induced PCOS mouse models [20,25,26,27,28,29]. Other agents such as melatonin, diacerein, and semaglutide (Table 1) have also been evaluated for their therapeutic effects [30,31,32]. These compounds have demonstrated the ability to improve ovarian morphology, restore regular estrous cycles, and normalize hormone levels by reducing testosterone, estradiol, and luteinizing hormone while increasing progesterone and follicle-stimulating hormone. Additionally, they improved insulin sensitivity, metabolic profiles, and body mass index (BMI) and reduced inflammation and oxidative stress. While none of these agents are known to directly engage with SIRT1, all of them resulted in SIRT1/AMPK pathway activation [20,25,26,27,28,29,30,31,32]. Collectively, these findings underscore the central role of the SIRT1/AMPK axis in PCOS pathophysiology. Although further mechanistic insights are needed, repurposing anti-inflammatory and anti-diabetic agents presents a promising strategy, particularly given the strong involvement of systemic inflammation and IR in PCOS.

In addition to pharmacological approaches, microRNA-based modulation of SIRT1 is also under investigation. miR-339-5p, associated with endothelial dysfunction in conditions like hypertension and cardiovascular disease, was found to be elevated in PCOS [33]. Inhibition of miR-339-5p increased SIRT1 expression and alleviated PCOS symptoms [33]. These findings highlight the role of post-transcriptional regulation of SIRT1 in PCOS, metabolic syndrome, and related cardiovascular conditions.

SIRT1 plays a crucial role in both conception and the maintenance of pregnancy in patients with PCOS. A recent study investigated the process of endometrial decidualization in PCOS, a key event characterized by the morphological transformation of endometrial mesenchymal cells into rounded, epithelioid-like cells in preparation for embryo implantation [34]. PDK4 is normally upregulated during this process. In PCOS, however, hyperandrogenism disrupts this process by inhibiting the SIRT1/AMPK/PDK4 signaling pathway and reduces the metabolic flexibility of endometrial tissue, thereby impairing decidualization and placentation and contributing to pregnancy complications. Notably, treatment of PCOS mouse models with either the SIRT1 activator SRT1720 or the AMPK activator A76 (Table 1) successfully promoted decidualization, further supporting the involvement of SIRT1 and AMPK in this pathway [34]. The widespread involvement of the SIRT1/AMPK axis across diverse physiological processes is both intriguing and compelling, warranting further investigation to validate these associations and elucidate their underlying mechanisms.

### 2.2. Other Sirtuins

SIRT2 is a known regulator of glucose metabolism, but its expression is downregulated in PCOS, contributing to IR [18,35]. Various compounds, including metformin, resveratrol, Dendrobium nobile-derived polysaccharides (DNPs), and nicotinamide mononucleotide (NMN) (Table 1) have been shown to upregulate or activate SIRT2, thereby restoring the expression of key enzymes involved in glucose metabolism, such as lactate dehydrogenase A (LDHA), pyruvate kinase M2 (PKM2), and hexokinase II (HK2) [18,21,35]. Beyond its role in glucose regulation, SIRT2 also contributes to the survival of granulosa cells (GCs). Specifically, SIRT2-mediated deacetylation of Dickkopf-1 (DKK1) promotes proliferation and inhibits apoptosis of KGN cells through the transforming growth factor β1 (TGF-β1)/Smad3 signaling pathway [36]. These studies indicate that SIRT2 modulation holds promise in alleviating IR and aiding in folliculogenesis.

Unlike SIRT1 and SIRT2, the mitochondrial sirtuins, SIRT3, SIRT4, and SIRT5, are, in some cases, upregulated in PCOS, likely as an adaptive response to oxidative stress and mitochondrial dysfunction in GCs [19]. SIRT3 mitigates oxidative stress by deacetylating FOXO1, which subsequently activates the FOXO1/peroxisome-proliferator-activated receptor-γ coactivator-1α (PGC-1α) pathway [37]. Treatments such as metformin, berberine, and the Chinese herbal medicine Bu-Shen-Tian-Jing Formula (BSTJF) have been shown to increase SIRT3 expression in GCs, alleviating PCOS symptoms in both KGN cells and PCOS animal models primarily by exerting an antioxidant effect [38,39,40].

**Table 1:** Summary of sirtuins in PCOS.

Sirtuin Isoform	Expression	Affected Pathways or Interactions	Pathological Outcomes	Therapeutic Intervention	Ref.
SIRT1	Downregulated	Downregulation of SIRT1-AMPK sxis	Hormonal dysregulation, oxidative stress, IR	Quercetin, resveratrol, and melatonin treatments activate SIRT1-AMPK axis	[17,20,23,24,25,26,27,28,29,30,31,32]
		Decreased SIRT1-mediated deacetylation of NF-κB	Inflammation, oxidative stress, metabolic dysregulation	Scoparone, sulforaphane, and diacerein treatments inhibit or induce degradation of NF-κB 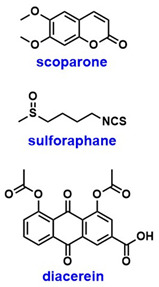	[17,20,25,26,27,28,29,31]
		Downregulation of PDK4	Impaired decidualization	SRT1720 (SIRT1 activator) and A76 (AMPK activator) restore decidualization ability 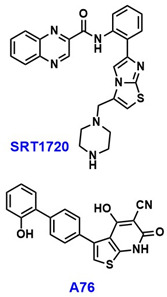	[17,34]
SIRT2	Downregulated	Decreased expression of LDHA, PKM2, Hk2	Impaired glucose metabolism	Metformin, resveratrol, DNPs, and NMN treatment upregulate SIRT2 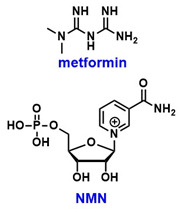	[18,21,35]
		Decreased SIRT2-mediated deacetylation of DKK1, downregulation of TGF-β1/Smad3 pathway	Diminished proliferation and increased cellular apoptosis		[36]
SIRT3	Upregulated	Increased SIRT3-mediated deacetylation of FOXO1, activation of FOXO1/PGC-1α pathway	Mitigation of oxidative stress	Metformin, berberine, and BSTJF treatments increase SIRT3 expression	[19,37,38,39,40]

## 3. Endometriosis (EM)

EM is a chronic inflammatory gynecological disorder characterized by the presence of endometrial-like tissue outside the uterus [41,42]. These ectopic tissues respond to monthly hormonal fluctuations, primarily estrogen and progesterone, leading to inflammation that can result in adhesions, scarring, internal bleeding, and infertility. EM affects approximately 10–15% of women of reproductive age worldwide, with estimates suggesting that at least one in nine women in the United States are affected [41,43]. Although the exact cause of EM remains unknown, several treatment options are available to manage disease progression and alleviate symptoms. These typically include surgical excision of lesions, hormonal therapies, and antioxidant agents [44]. Importantly, EM is now recognized as a systemic disease, associated with increased risks for other health conditions such as cardiovascular disease, asthma, certain autoimmune disorders, and ovarian and breast cancers—highlighting its significant impact on women’s long-term health [45].

SIRT1, SIRT2, and SIRT7 have been reported to be upregulated in endometriotic tissues (Table 2) [46,47,48,49,50]. SIRT1 has been implicated in the progression of EM, particularly in regulating autophagy and contributing to progesterone resistance [51]. In contrast, SIRT3 has demonstrated protective effects against reactive oxygen species (ROS) within the mitochondrial matrix of ovarian endometriomas [52].

### 3.1. SIRT1

The expression of SIRT1 in EM has been variably reported; however, it is generally accepted that SIRT1 is upregulated in ectopic endometrial tissues [47,48,49,50]. In contrast, a decrease in SIRT1 expression was observed in eutopic endometrial tissues [53], while another study reported no significant difference between eutopic and ectopic endometrial tissues [54]. SIRT1 overexpression was identified in the blood serum of patients with stage III/IV EM, suggesting its potential as a biomarker for advanced disease. Interestingly, a decrease in serum SIRT1 levels was also reported in patients with stage I/II EM, indicating a possible stage-dependent pattern of SIRT1 expression [55].

It has been demonstrated that elevated SIRT1 levels can overcome damage-induced senescence in EM tissues by inhibiting both the p53 and p38 MAPK pathways [48]. This senescence escape facilitates the epithelial–mesenchymal transition (EMT), where epithelial cells lose their identity and acquire mesenchymal characteristics, enhancing tissue invasiveness [48]. In a subsequent study, it was reported that METTL3, a key component of the m6A methyltransferase complex, directly interacts with SIRT1 and promotes m6A modification, thereby suppressing SIRT1 transcription in normal endometrial tissues [49]. However, in ectopic lesions, METTL3 is downregulated, leading to increased SIRT1 expression. In a grafted EM tissue mouse model, larger lesion sizes were observed in mice with overexpressed SIRT1, further supporting the role of SIRT1 in the progression of EM [49].

Excessive iron accumulation in endometriotic lesions was identified to induce upregulation of SIRT1, which in turn triggered a protective autophagic response and a reduction in cell proliferation within EM tissues [56]. A negative correlation between SIRT1 and FOXO-1 was observed in EM, attributed to the SIRT1-mediated deacetylation and subsequent degradation of FOXO-1 [53]. It was further demonstrated that FOXO-1 expression was positively associated with autophagy-related proteins such as Beclin-1, Atg5, and Atg7, implicating the SIRT1/FOXO-1 signaling pathway in the regulation of autophagy and the progression of EM [53]. In a mouse model of EM, treatment with Bushen Wenyang Huayu Decoction (BWHD) was shown to restore SIRT1 protein and mRNA levels, attenuate autophagy, and result in significant reductions in the weight and volume of ectopic lesions [53].

Progesterone resistance in endometrial tissues has been linked to SIRT1, both in eutopic and ectopic endometrium [48,50,57]. In healthy samples, SIRT1 expression is observed during menstruation, potentially as a response to inflammation, before subsiding to permit progression through the menstrual cycle [50]. However, due to the chronic inflammation characteristic of EM, SIRT1 is overexpressed throughout all stages of the menstrual cycle in EM tissues. In a SIRT1 overexpression mouse model, increased SIRT1 levels were shown to suppress the expression of progesterone target genes through a direct protein–protein interaction between SIRT1 and progesterone receptors (PGRs), resulting in progesterone resistance [50].

### 3.2. SIRT3

A significant reduction in SIRT3 expression in endometrioma tissues has been identified, along with decreased activity of known SIRT3 substrates: succinate dehydrogenase (SDH), glutamate dehydrogenase (GDH), and manganese superoxide dismutase (MnSOD) [52]. SIRT3 is known to regulate the cellular response to ROS through the deacetylation of SDH, GDH, and MnSOD [58]. Therefore, the downregulation of SIRT3 may be responsible for the reduced activity of these enzymes and the subsequent decline in total antioxidant capacity of EM tissues, which reflects the diminished ability of cells to neutralize ROS [52].

**Table 2:** Summary of sirtuins in endometriosis.

Sirtuin Isoform	Expression	Affected Pathways or Interactions	Pathological Outcome	Therapeutic Intervention	Ref.
SIRT1	Upregulated	SIRT1 downregulates the p53/p38 MAPK pathway through deacetylation of p53	Facilitation of EMT Enhanced tumor invasiveness		[48]
		SIRT1/PGR direct interaction suppresses the expression of progesterone target genes	Progesterone resistance		[48,50,57]
	Downregulated	SIRT1-mediated deacetylation leads to degradation of FOXO-1	Upregulation of autophagy-related proteins	BWHD Treatment restores SIRT1 levels and attenuates autophagy	[53]
SIRT3	Downregulated	Reduction in SIRT3-mediated deacetylation and activation of ROS regulating proteins	Decreased ability to neutralize ROS		[52,58]

## 4. Preeclampsia (PE)

PE is a serious pregnancy complication associated with significant fetal and maternal morbidity and mortality. It affects approximately 2–8% of pregnancies and is clinically defined by new-onset hypertension during pregnancy [59]. PE is further characterized by placental oxidative stress, an exaggerated inflammatory response, inadequate extravillous trophoblast (EVT) invasion, and premature placental senescence [60,61,62,63,64,65]. Emerging evidence suggests that sirtuins, particularly SIRT1–3, SIRT5, and SIRT6, may contribute to the pathophysiology of PE by modulating these key processes (Table 3).

### 4.1. SIRT1

Decreased levels of SIRT1 have been reported in both the serum and placenta of PE patients and PE mouse models [59,61,63,65,66,67]. PE symptoms, like hypertension, proteinuria, fetal growth restriction, and kidney injury, were observed in SIRT1-knockdown mice, and these symptoms improved upon administration of SRT2104 (Table 3), an allosteric activator that binds to the N-terminus of SIRT1 [66,68]. Furthermore, several SIRT1 variants including rs7895833, rs7069102, and rs2273773 were seen to affect SIRT1 promoter activity. These variants were also associated with increased oxidative stress and were identified as risk factors for PE development [69].

Reduced SIRT1 expression has been associated with placental senescence, a hallmark of PE. For example, 4-hydroxy-2-nonenal (HNE), a lipid-peroxidation-derived aldehyde elevated in preeclamptic placentas, has been shown to form adducts with SIRT1, thereby reducing its enzymatic activity and contributing to premature placental senescence [64]. Telomere shortening, another marker of cellular senescence, is also associated with PE. Notably, the SIRT1 variant rs12778366 has been linked to telomere shortening, further supporting the role of SIRT1 in the pathogenesis of placental senescence in PE [70]. In addition, microRNA miR-494 and miR-199a-5p, which are upregulated in PE, have been shown to inhibit SIRT1 expression and promote cellular senescence [63,71].

SIRT1 deficiency is associated with impaired invasiveness of EVTs, contributing to decreased placental perfusion, a hallmark of PE [59,62]. SIRT1 regulates the glycoprotein CD74, and its downregulation leads to diminished trophoblast invasiveness. Notably, activation of SIRT1 has been shown to restore trophoblast proliferation and invasion in CD74-knockdown models, suggesting that SIRT1 may influence PE pathophysiology through modulation of CD74 [62]. SIRT1 also regulates the Sonic Hedgehog (SHH) signaling pathway, which is negatively impacted by oxidative stress in preeclamptic placental tissues, indicating a potential protective role of SIRT1 via SHH signaling [67]. Furthermore, SIRT1 levels are positively correlated with placental growth factor (PlGF) and negatively correlated with soluble FMS-like tyrosine kinase-1 (sFlt-1), an antiangiogenic factor elevated in PE, further linking SIRT1 deficiency to disease progression [59]. In addition, alpinumisoflavone (AIF) (Table 3), a prenylated isoflavonoid derived from the mandarin melon berry (*Cudrania tricuspidata*), has been reported to protect trophoblast cells from oxidative stress by activating SIRT1 [61]. Collectively, SIRT1 deficiency may contribute to multiple pathological features of PE—including oxidative stress, premature placental senescence, and inadequate EVT invasion—through diverse mechanisms involving CD74, SHH signaling, and telomere regulation.

### 4.2. SIRT2

Both early- and late-onset PE patients have been shown to exhibit significantly reduced serum levels of SIRT2 compared to healthy controls [72]. Overexpression of SIRT2 has been found to enhance trophoblast motility, suggesting a potential protective role in placental development. Reduced SIRT2 expression is also associated with decreased levels of mitochondrial biogenesis-related proteins, including PGC1α, NRF1, and TFAM, indicating one possible mechanism by which SIRT2 may influence PE pathophysiology [73]. Another potential pathway involves the regulation of atypical chemokine receptor 2 (ACKR2). SIRT2 deacetylates the transcription factor p65, which leads to suppression of the microRNA miR-146a [74]. Downregulation of miR-146a, in turn, upregulates ACKR2, a receptor whose overexpression has been shown to enhance trophoblast invasion and migration. Thus, diminished SIRT2 levels may contribute to the development of PE by reducing ACKR2 expression and impairing trophoblast function [74].

### 4.3. SIRT3

Downregulation of SIRT3 has been observed in both placental tissue and serum from patients with PE [75,76]. Meanwhile, overexpression of SIRT3 has been shown to enhance trophoblast migration and proliferation, suggesting a protective role in placental function [76]. One mechanism by which SIRT3 may modulate PE is through the regulation of MnSOD. SIRT3 activates MnSOD via deacetylation, thereby reducing mitochondrial oxidative stress. Notably, MnSOD overexpression has been shown to promote trophoblast migration even in the context of SIRT3 deficiency, indicating that reduced SIRT3 levels may contribute to PE by limiting MnSOD activation [75]. Another potential mechanism involves necroptosis. Elevated levels of necroptosis biomarkers such as receptor-interacting protein kinase 1 (RIPK1), RIPK3, and phosphorylated mixed lineage kinase domain-like protein (p-MLKL) have been reported in PE placentas [76]. These markers are inversely correlated with SIRT3 expression: SIRT3 knockdown increases their levels, while SIRT3 overexpression suppresses them [76]. Thus, SIRT3 may confer protection against PE by mitigating both oxidative stress and necroptotic cell death.

### 4.4. SIRT5

SIRT5 is believed to contribute to the pathophysiology of PE through its regulation of protein kinase AMP-activated catalytic subunit alpha 2 (PRKAA2) succinylation [77]. PRKAA2 expression is elevated in the placental tissue of PE patients, and reduced SIRT5 levels lead to increased succinylation of PRKAA2. Overexpression of PRKAA2 has been shown to promote apoptosis in placental cells, and similarly, SIRT5 knockdown also induces placental cell apoptosis [77]. These findings suggest that SIRT5 may protect against PE by limiting PRKAA2 succinylation and preventing trophoblast apoptosis.

### 4.5. SIRT6

Placental tissues from PE patients exhibit elevated levels of oxidative stress and iron accumulation [78]. SIRT6 has been shown to mitigate oxidative stress and ferroptosis through activation of the Nrf2/HO-1 pathway, which involves antioxidant enzymes that regulate iron metabolism [78]. Given its role in modulating both oxidative stress and ferroptosis, SIRT6 may contribute to the pathogenesis of PE.

**Table 3:** Summary of sirtuins in PE.

Sirtuin Isoform	Expression	Affected Pathways or Interactions	Pathological Outcome	Therapeutic Intervention	Ref.
SIRT1	Downregulated	SIRT1 suppresses NF-κB activated by CD74	Decreased EVT invasion	SIRT1 activators: 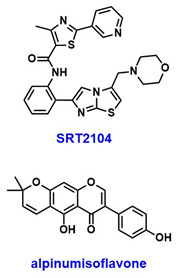	[59,61,62,66,67]
		SIRT1 deacetylates and activates transcription factors that activate the SHH pathway	Increased oxidative stress		
		SIRT1 suppresses hypoxia-driven sFlt-1 release while upregulating PIGF expression	Reduction in placental growth due to impaired angiogenesis		
SIRT2	Downregulated	Reduced SIRT2 mediated stabilization of PGC1α/ NRF1/TFAM signaling	Dysfunction of mitochondrial biogenesis		[73]
		SIRT2 indirectly regulates the transcription of ACKR2	Reduced trophoblast motility		[74]
SIRT3	Downregulated	Reduced deacetylation and activation of MnSOD	Reduced trophoblast motility		[75]
SIRT5	Downregulated	Increased succinylation of PRKAA2	Apoptosis of placental cells		[77]
SIRT6	Not reported	SIRT6 deacetylates NRF2 and promotes NRF2/HO-1 pathway	Dysregulation of SIRT6 may increase oxidative stress and ferroptosis		[78]

## 5. Gestational Diabetes Mellitus (GDM)

GDM is defined as diabetes first diagnosed during the second or third trimester of pregnancy. In GDM, pancreatic β-cells fail to adequately respond to the increased insulin demand associated with pregnancy, resulting in maternal hyperglycemia. GDM shares several risk factors with PE, including obesity, advanced maternal age (over 35), and multiple gestations [70]. It is also linked to placental hypoxia and premature senescence of human umbilical cord endothelial cells [79,80]. SIRT1, SIRT6, and SIRT7 have been implicated in the pathophysiology of GDM.

### 5.1. SIRT1

SIRT1 expression is reduced in both the blood and placental tissues of patients with GDM, as well as in trophoblast cell models of the disease [80,81,82]. Overexpression of SIRT1 has been shown to enhance glucose uptake and improve the viability of trophoblast cells. In GDM, key components of the insulin signaling pathway such as phosphorylated IRS1 (p-IRS1) and AKT (p-AKT) are upregulated, while levels of suppressor of cytokine signaling 3 (SOCS3) are decreased [81]. SIRT1 is thought to modulate GDM by reducing the phosphorylation and acetylation of signal transducer and activator of transcription 3 (STAT3), thereby downregulating SOCS3. This reduction in SOCS3 subsequently increases the phosphorylation of IRS1 and AKT, consistent with observations in GDM trophoblast cells [81].

SIRT1 is downregulated under hypoxic conditions, further supporting its reduced expression in GDM, where placental hypoxia is a well-recognized feature. This hypoxia-induced downregulation of SIRT1 is associated with decreased levels of FK506-binding protein-like (FKBPL), a regulator of glucocorticoid receptor signaling through the HSP90, CD44, and Notch pathways [80]. Additionally, SIRT1 is believed to upregulate peroxisome proliferator-activated receptor gamma (PPARγ), a transcription factor involved in inflammation and IR, by deacetylating QKI5 and subsequently activating the PI3K/AKT pathway, thereby exerting a protective effect against GDM [83].

Activation of SIRT1 has been shown to alleviate symptoms of GDM [83,84]. In rat models, the SIRT1 activator SRT1720 (Table 4) has been reported to improve abnormal glucose metabolism and reduce lung tissue injury associated with GDM [83]. Additionally, mogroside V (Table 4), the primary bioactive compound in *Siraitia grosvenorii*, a traditional Chinese medicinal plant, has been shown to upregulate SIRT1 expression and mitigate placental and pancreatic damage in GDM rat models [84]. In contrast, mouse studies have demonstrated that exposure to polystyrene microplastics inhibits hepatic SIRT1 and induces glucose dysregulation during pregnancy [85].

### 5.2. SIRT6

SIRT6 is downregulated in the vascular walls of patients with type 2 diabetes mellitus (T2DM) and GDM. This downregulation may contribute to the increased risk of atherosclerotic cardiovascular disease (ASCVD) observed in diabetic patients, potentially through regulation of Caveolin-1 [86]. During the early stages of atherogenesis, Caveolin-1 facilitates the transcytosis of low-density lipoprotein (LDL) across endothelial cells, leading to its accumulation in the subendothelial space. Notably, Caveolin-1 is upregulated in the vascular walls of both T2DM and GDM patients. SIRT6, along with SIRT1, has been shown to interact with Caveolin-1, modulating its acetylation status and potentially influencing its activity [86].

### 5.3. SIRT7

SIRT7 expression is elevated in the placental tissue of patients with GDM, alongside increased levels of oxidative stress markers [87]. A positive correlation has also been observed between SIRT7 expression and both BMI and triglyceride levels. Emerging evidence suggests that the microRNA miR-125b-5p may regulate SIRT7 expression in GDM with downregulation of miR-125b-5p in the placentas of GDM patients suppressing SIRT7 expression.

**Table 4:** Summary of sirtuins in GDM.

Sirtuin Isoform	Expression	Affected Pathways or Interactions	Pathological Outcome	Therapeutic Intervention	Ref.
SIRT1	Downregulated	SIRT1 deacetylatesSTAT3 and regulates STAT3/SOCS3/IRS-1/AKT pathway	Insulin signaling pathway dysfunction	SRT1720: SIRT1 activator Mogroside V: Upregulates SIRT1 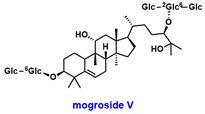	[80,81,83,84]
		SIRT1 positively regulates transcription of FKBPL and modulates FKBPL/HSP90/CD44/Notch pathway	Glucocorticoid receptor signaling dysfunction		
		SIRT1 activates PPARγ/QKI5/PI3K/AKT pathway by deacetylating QKI5 and promoting PPARγ expression	Inflammation and insulin resistance		
SIRT6	Downregulated	SIRT6 modulates the acetylation status of Caveolin-1	Accumulation of LDL in vascular walls		[86]
SIRT7	Upregulated	-	High BMI and TG levels		[87]

## 6. Postpartum Depression (PPD)

PPD is a mood disorder characterized by symptoms such as depressed mood, agitation, sleep disturbances, and, in severe cases, suicidal ideation [88]. These symptoms typically arise during pregnancy or within four weeks after delivery and affect approximately 7–15% of individuals during the perinatal period. PPD not only impacts maternal well-being but also negatively affects infant development and bonding. SIRT1 has been implicated in the pathophysiology of PPD, as lower SIRT1 expression has been observed in mouse models of the condition [89]. Treatment of these models with SIRT1 activators such as resveratrol and SRT2104 significantly alleviated depressive symptoms, further supporting a potential role for SIRT1 in the development and treatment of PPD [89,90].

## 7. Fertility

Sirtuins, particularly SIRT1, have been associated with female fertility. In humans, infertility has been linked to reduced serum levels of SIRT1 [91]. Similarly, in cattle, a single-nucleotide polymorphism in the promoter region of the *SIRT1* gene is significantly associated with reproductive traits such as calving interval and days open [92]. While sirtuins appear to play an important role in fertility, it is a complex and multifactorial process influenced by numerous physiological, genetic, and environmental factors. Certain conditions, such as EM and PCOS, are strongly associated with infertility; however, these are discussed in detail in other sections of this review and will not be addressed further here. For the sake of clarity, this section will focus specifically on the roles of sirtuins in the ovaries, uterus, and oocytes as they relate to female fertility (Table 5).

### 7.1. Ovaries

One key factor affecting ovarian-related fertility is diminished ovarian reserve (DOR), which involves both a quantitative and qualitative decline in ovarian follicles. DOR is a hallmark of ovarian aging and is closely associated with reduced fertility potential [93,94]. Sirtuins have been implicated in this process, as significant differences in the expression of proteins involved in sirtuin signaling pathways have been observed in aged mouse ovaries [93]. Specifically, reduced SIRT1 expression has been reported in cumulus granulosa cells of patients with DOR. This reduction may contribute to mitochondrial dysfunction, a characteristic feature of DOR [94].

Another ovarian-related factor influencing fertility is premature ovarian insufficiency (POI), defined as the decline in or loss of ovarian function before the age of 40 [95]. Mitochondrial dysfunction is a well-established risk factor for POI, which is also associated with oxidative stress and DNA damage, particularly following chemotherapy [96]. In mouse models of POI, SIRT1 mRNA expression is significantly reduced [97]. Combination therapy using pyrroloquinoline quinone (PQQ, Table 5), an antioxidant known to enhance fertility, and mitochondria derived from mesenchymal stem cells (MSC-Mito) has been shown to upregulate SIRT1 expression in both POI mouse models and KGN cell lines. This treatment also reduces apoptosis in KGN cells, suggesting that PQQ and MSC-Mito may help mitigate POI, at least in part, through SIRT1-mediated mechanisms [97].

### 7.2. Uterus

Sirtuins have been implicated in uterine aging, a process that contributes to diminished fertility. In senescence-induced primary human endometrial cells, the expression of both SIRT1 and SIRT6 is reduced [98]. Bushen Jianpi Tiaoxue Decoction (BJTD), a traditional Chinese medicine, has been shown to alleviate oxidative stress and apoptosis associated with uterine aging while also upregulating SIRT1 expression [99]. These findings suggest that SIRT1 may be a key target of BJTD, contributing to its protective effects against uterine aging.

Implantation is a critical step in the initiation of pregnancy and involves decidualization. In SIRT1-knockout mice, the number of implantation sites is significantly reduced, and defects in decidualization have been observed [100]. These effects may be mediated through the dysregulation of PGR and FOXO1, both of which are downregulated in the absence of SIRT1 [100].

### 7.3. Oocyte

Postovulatory aging (POA) occurs when an oocyte is not fertilized within 24 h of ovulation in humans. It is characterized by degeneration of the first polar body, enlargement of the perivitelline space, accumulation of ROS, energy depletion, and chromosomal and DNA damage [101]. POA leads to a decline in oocyte quality and is associated with reduced fertilization success rates. In murine models, treatment with nicotinamide riboside (NR) has been shown to increase SIRT1 expression, restore depleted NAD^+^ levels, and reduce both DNA damage and ROS accumulation—effects that may enhance fertilization outcomes [102].

Chemotherapeutic agents such as doxorubicin (DXR) have been shown to impair fertility and are associated with oxidative damage and meiotic failure in oocytes [103]. SIRT1 may play a role in this process, as its expression is decreased in the oocytes of mice exposed to DXR. Administration of resveratrol, a known SIRT1 activator, has been shown to reduce ROS accumulation and mitigate meiotic defects, thereby improving oocyte quality and supporting embryonic development [103].

**Table 5:** Summary of sirtuins relating to fertility.

Sirtuin Isoform	Affected Organ/Cells	Expression	Outcome	Therapeutic Intervention	Ref.
SIRT1	Ovaries	Downregulated	DOR and POI	Treatment with PQQ or MSC-mito 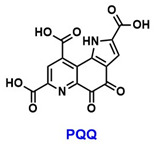	[94,97]
	Uterus	Downregulated	Defects in decidualization	Treatment with BJTD	[99,100]
	Oocytes	Downregulated	POA	Treatment with resveratrol or NR to restore SIRT1 expression and activity	[102,103]

## 8. Cancer

### 8.1. Cervical Cancer

**Figure 1:**
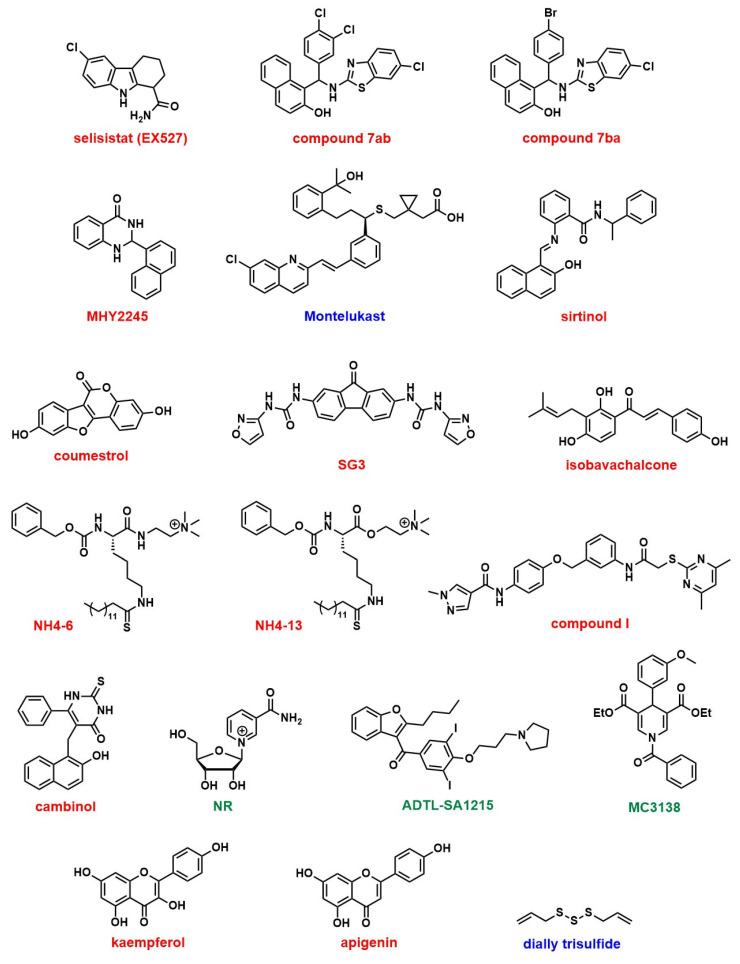
Compounds studied in cervical, breast, and ovarian cancers. Sirtuin inhibitors are highlighted in red, activators in green, and other agents in blue.

Sirtuins play diverse and critical roles in cervical cancer progression, with each isoform contributing to distinct molecular pathways (Table 6). Among these, SIRT1 is the most extensively studied and has been suggested both as a tumor promoter and contributor of therapy resistance. In cervical cancer, reprogrammed NAD^+^ metabolism activates SIRT1 to promote transcription of PD-L1 expression through histone deacetylation. SIRT1 also deacetylates PD-L1 and increases its nuclear localization. contributing to immune evasion. Inhibition of SIRT1 using EX527 (Figure 1) reduces PD-L1 nuclear translocation, suppresses tumor growth, and enhances the efficacy of anti-PD-L1 immunotherapy [104]. Furthermore, the MALAT1/miR-124/SIRT1 axis regulates pyroptosis, while transcription factor TCF3 activates SIRT1 to trigger Wnt/β-catenin signaling and tumor growth [105].

SIRT1 activity is closely associated with human papillomavirus (HPV) oncogenesis. HPV16 E2 interacts with TopBP1 to disrupt SIRT1 function during mitosis, leading to acetylation and stabilization of p53 and CHK2, which in turn promotes DNA damage response and viral persistence [106]. Additionally, SIRT1 facilitates E2 protein degradation in the absence of TopBP1, driving viral genome integration and cancer progression [107]. Clinically, SIRT1 level increases with disease severity, from low-grade cervical intraepithelial neoplasia (CIN) to invasive squamous cell carcinoma [108]. This upregulation is partly driven by HPV16 E7, which boosts SIRT1 protein levels and promotes tumor proliferation, invasion, and survival [109]. Therapeutically, SIRT1 inhibition restores p53 function and sensitizes tumors to cisplatin, reducing tumor volume by 50% in mouse models [110]. In parallel, nicotinamide suppresses the SIRT1–CD38–EMT axis, reducing EMT markers by 40% and increasing apoptosis by 35% in HPV-positive cells [111].

SIRT3 is downregulated in cervical cancer due to ALKBH5-mediated m6A RNA modification, which disrupts fatty acid metabolism through inhibition of ACC1 [112]. This reduction in SIRT3 is associated with enhanced tumor growth and poor prognosis. Similarly, SIRT4 acts as a tumor suppressor by regulating glutamine metabolism. The PIK3CA-E545K mutation suppresses SIRT4 via EP300, leading to increased glutamine utilization, proliferation, and radioresistance. Pharmacological inhibition of PIK3 restored radiosensitivity by targeting this axis [113]. Independently, SIRT4 also inhibits the MEK/ERK/c-Myc pathway, reducing glutaminase expression and impeding proliferation and metastasis, reinforcing its anti-tumor role [114].

In contrast, SIRT7 is overexpressed in cervical squamous cell carcinoma and promotes tumor growth through a positive feedback loop with USP39 and FOXM1, which regulates autophagy and oxidative stress [115]. Moreover, SIRT7 is phosphorylated by ATM during DNA damage, impairing mismatch repair through MSH2 degradation, increasing mutation frequency by two-fold, and contributing to chemoresistance and genomic instability [116].

### 8.2. Breast Cancer

The sirtuin family has emerged as a key player in the regulation of breast cancer, impacting various aspects such as tumor initiation, progression, metastasis, metabolism, and treatment response. The seven mammalian sirtuins (SIRT1–7) have diverse functions based on their cellular context, subcellular localization, and tumor subtype, positioning them as potential biomarkers and therapeutic targets (Table 6).

#### 8.2.1. SIRT1

SIRT1 is frequently overexpressed in breast cancer, particularly in patients with metastatic disease. SIRT1 participates in DNA repair mechanisms. Its elevated expression correlates with decreased PP4 phosphatase levels, impairing DNA damage response and facilitating tumor progression [117]. Serum SIRT1 levels are significantly elevated in breast cancer compared to benign fibroadenoma cases and healthy controls, indicating its potential as a diagnostic biomarker [118,119]. The regulatory axis involving long non-coding RNA (lncRNA) PVT1 and miR-146a modulating SIRT1 expression further supports its utility as a non-invasive biomarker [118]. SIRT1’s functional impact appears highly dependent on its subcellular localization and the tumor’s molecular subtype. In Saudi breast cancer patients, high nuclear SIRT1 correlated with lymph node metastasis and poor disease-specific survival in HER2+ tumors, while cytoplasmic SIRT1 had opposing effects [120]. Conversely, luminal A tumors with elevated nuclear SIRT1 demonstrate favorable outcomes, illustrating subtype-dependent duality [120].

Benzothiazole derivatives (7ab and 7ba) (Figure 1) that inhibit SIRT1, SIRT2, and SIRT3 in MCF-7 breast cancer cells reduced cell proliferation with IC_50_ values of 11.4 µM and 9.6 µM. These inhibitions led to increased acetylation and activation of p53, which triggered both apoptosis and autophagy [121]. MHY2245 (Figure 1) also inhibits SIRT1 and induces apoptosis via p53 in breast cancer [122].

Montelukast (Figure 1) was shown to suppress EMT and angiogenesis in triple-negative breast cancer (TNBC) through inhibition of the SIRT1/AKT signaling axis, reducing SIRT1 and vimentin expression while upregulating E-cadherin. These effects were potentiated by co-administration with the SIRT1 inhibitor sirtinol (Figure 1), reinforcing SIRT1 as a therapeutic target to disrupt EMT and metastasis [123].

Estrogen-related receptor β (ERRβ) enhances SIRT1 expression in ER-positive breast cancer cells, which amplifies ER and GPER signaling to promote oncogenesis, proliferation, and chemoresistance, which were reversed by SIRT1 inhibition [124]. The selective SIRT1 inhibitor selisistat (EX527) (Figure 1) enhanced paclitaxel cytotoxicity and increased apoptosis through modulation of the SIRT1–AKT/S1PR1/tubulin pathway [125]. The dietary phytochemical coumestrol (Figure 1), a known inhibitor of SIRT1–3, demonstrated significant reduction in cell proliferation and colony formation in MCF-7 and MDA-MB-231 breast cancer cells, accompanied by decreased SIRT1 expression and elevated intracellular ROS levels.

#### 8.2.2. SIRT2

SIRT2, primarily cytoplasmic and associated with microtubule regulation, modulates breast cancer cell migration, chemotherapy response, and cell cycle progression. Radiotherapy downregulates SIRT2 levels in breast cancer patients post-treatment, with pre-radiotherapy SIRT2 levels positively correlated with treatment response, suggesting SIRT2 as a potential predictive biomarker [126].

SIRT2 inhibition through sirtinol co-delivered with doxorubicin in nanomicelles (DOX@LPS-PEC) reduced TNBC cell migration by approximately 50%, suppressed IL-6 secretion, and enhanced ROS generation, disrupting the activities of TLR4 and SIRT2 and improving chemotherapy efficacy [127]. Selective SIRT2 inhibitors such as SG3 (IC_50_ = 1.95 µM) and isobavachalcone (IC_50_ = 0.84 µM) (Figure 1) demonstrated strong antitumor activity by inducing α-tubulin hyperacetylation, suppressing the STAT3/c-Myc and Snail/MMP pathways, resulting in apoptosis and S-phase arrest in TNBC models, with significant tumor size reduction [128,129]. Moreover, SIRT1 and SIRT2 post-translationally regulate the activity of arylamine N-acetyltransferase 1 (NAT1), which is overexpressed in in breast cancer cells, where inhibition of SIRT1 and 2 reduced NAT1 enzymatic function, potentially impacting tumor growth, suggesting their potential as therapeutic targets [130].

Pan-SIRT1–3 inhibitor NH4-6 and SIRT2-selective inhibitor NH4-13 (Figure 1) both suppressed tumor growth, but NH4-13 had lower toxicity and comparable efficacy in a breast cancer model [131]. Another study shows that combining a SIRT2-selective inhibitor (compound I) (Figure 1) with sorafenib enhanced tumor growth inhibition through increased apoptosis and G0/G1 arrest [132]. Contrarily, the SIRT1/2 inhibitor cambinol (Figure 1) showed antagonistic interaction with paclitaxel in TNBC cells, reducing caspase-3 activation and chemotherapy efficacy. These studies emphasize the need for isoform-specific targeting and careful pharmacological combinations [133].

#### 8.2.3. SIRT3

SIRT3, one of the mitochondrial sirtuins, plays multifaceted roles in breast cancer metabolism and progression. High SIRT3 expression in TNBC patients has been associated with poor overall survival and chemoresistance. Notably, patients who achieved pathological complete response exhibited significantly lower SIRT3 expression, with a hazard ratio of 17.98 for survival—highlighting a paradoxical, context-dependent role of SIRT3 in breast cancer [134].

Genetic studies associate a variable number tandem repeat (VNTR) polymorphism (0R/0R genotype) in SIRT3 intron 5 with a significant increased breast cancer risk, implicating mitochondrial dysfunction in tumor susceptibility [135]. Additionally, SIRT3 maintains mitochondrial homeostasis by post-translationally modifying MnSOD at lysine 68 (K68Ac). Hyperacetylation at this site, regulated by SIRT3, shifts MnSOD from its functional superoxide-scavenging tetrameric form to a peroxidase-active monomer, thereby impairing mitochondrial function and promoting chemoresistance to cisplatin and doxorubicin in ER-positive breast cancer [136].

SIRT3 also exerts tumor-suppressive effects through activation by PGC-1α, which enhances oxidative phosphorylation while inhibiting glycolysis and cellular proliferation, an activity correlated with improved patient survival [137]. Activation of SIRT3 by nicotinamide riboside (NR) (Figure 1) induced antioxidant enzymes such as MnSOD2 and NRF2, protecting against paclitaxel-induced peripheral neuropathy [138]. A novel SIRT3-selective activator compound 33c (ADTL-SA1215) (Figure 1) enhances autophagy and mitophagy, reducing proliferation and migration in vitro and in vivo, suggesting a therapeutic potential [139]. Conversely, pharmacological activation of SIRT3 under acidic extracellular pH promotes mitochondrial carbonic anhydrase VB (CAVB) activation and autophagy, supporting cell survival in harsh tumor microenvironments [140].

#### 8.2.4. SIRT5

SIRT5, a mitochondrial lysine deacylase, influences breast cancer metabolism by modulating glutamine metabolism, ROS accumulation, and autophagy/mitophagy [141]. Activation of SIRT5 by MC3138 (Figure 1) reduced glutamine metabolism, particularly in metabolically active TNBC [142]. Combination treatment of MC3138 with lanthanum acetate, a phosphate chelator, further decreases cell viability by modulating autophagy and mitophagy pathways, which leads to an accumulation of cytosolic and mitochondrial ROS, contributing to cytotoxicity [142]. However, high SIRT2 and SIRT5 expression also correlated with drug resistance profiles across multiple breast cancer lines and chemotherapies, underscoring their roles in chemoresistance mechanisms [143].

#### 8.2.5. SIRT6

SIRT6 has been implicated as a pro-metastatic factor, particularly in HER2-positive breast cancer [144]. Although initial overexpression delays tumor onset, it eventually enhances mammosphere formation and metastasis, promoting invasive phenotypes. Mechanistically, SIRT6 repressed the tumor suppressor TBX3 by deacetylating histone H3 at its promoter, leading to enhanced metastatic capacity [144]. Clinically, SIRT6 amplification correlated with HER2 co-amplification and poor relapse-free survival [144]. Moreover, SIRT6 promoted breast cancer metastasis by upregulating matrix metalloproteinase-9 (MMP-9) through the MAPK, NF-κB, and AP-1 pathways; silencing SIRT6 reduced cell invasion and migration in MCF-7 and MDA-MB-231 cells, suggesting its direct role in metastatic progression [145].

Natural flavonoids such as kaempferol and apigenin (Figure 1) inhibit SIRT3 and SIRT6 in TNBC, reducing stem-like features by decreasing mammosphere formation and inducing cell cycle arrest in S-phase, illustrating epigenetic and metabolic disruption as therapeutic strategies [146]. Nutritional ketosis via β-hydroxybutyrate reprogrammed sirtuin expression and gene methylation in TNBC and obese individuals, upregulating SIRT1, SIRT3, and SIRT6, restoring tumor suppressor expression, and downregulating oncogenes, demonstrating metabolic intervention potential [147].

#### 8.2.6. SIRT7

SIRT7 expression is elevated in the early stages of breast cancer, where it promotes tumor progression by enhancing cell proliferation and migration. In contrast, its levels decline in metastatic tumors, suggesting a functional shift as the disease advances [148]. While some studies report that SIRT7 depletion suppresses proliferation and migration through activation of the p38 MAPK pathway, others show that loss of SIRT7 accelerates tumor growth in mouse models, underscoring its context-dependent role [148].

At later stages, elevated SIRT7 may suppress metastasis by deacetylating SMAD4, leading to its degradation and subsequent inhibition of TGF-β signaling, thereby limiting epithelial–mesenchymal transition and metastatic spread [148]. SIRT7 also counteracts metastasis in doxorubicin-resistant breast cancer cells by epigenetically repressing the tyrosine kinase receptor TIE2/TEK via H3K18 deacetylation at its gene promoters, which would otherwise promote migration [149]. Reduced SIRT7 disrupts its interaction with LAP2α, accelerating LAP2α degradation, increasing chromosomal instability (CIN), and facilitating metastatic progression [150]. Moreover, elevated SIRT7 expression correlates with aggressive tumor phenotypes and poor patient prognosis, positioning it as a promising therapeutic target [151]. Collectively, these findings illustrate the dualistic nature of SIRT7 in breast cancer: acting as a tumor promoter in early disease but potentially serving as a metastasis suppressor in advanced stages.

### 8.3. Uterine and Endometrial Cancer

Uterine cancer (UC) is the most common type of gynecological cancer among American women and ranks as the fourth most common cancer overall in this population. It is estimated to account for 3.4% of new cancer cases and 2.2% of all cancer-related deaths in the United States in 2025 [152]. UC refers to any abnormal growth of cells within the uterus, whereas endometrial cancer (EC) is a more specific term referring to cancer originating from the endometrium, the inner lining of the uterus. EC is further classified into two main subtypes: Type I (endometrioid) and Type II (non-endometrioid). Endometrioid tumors account for approximately 75–80% of UC cases and are characteristically estrogen-sensitive [153]. Type I tumors are typically detected at earlier stages and therefore tend to have a more favorable prognosis. In contrast, non-endometrioid tumors, which include uterine serous carcinomas and uterine sarcomas, are less common, generally more aggressive, and associated with a poorer prognosis [153,154]. SIRT1, SIRT2, and SIRT7 have been implicated in the development and metastasis of EC, while SIRT6 has been shown to exhibit anti-tumor properties in this context (Table 6).

#### 8.3.1. SIRT1

In recent years, SIRT1 has been implicated as both a tumor suppressor and a tumor promoter, depending on the cellular and molecular context. In EC research, findings have varied depending on the histological subtype examined. SIRT1 expression was positively correlated with the prognostic markers ARID1A and β-catenin and also associated with improved overall survival and longer progression-free survival for endometrioid and clear-cell EC [155]. In contrast, no significant relationship between SIRT1 expression and progression-free survival was reported in non-endometrioid EC [156].

SIRT1 knockdown has been shown to inhibit the proliferation, migration, and invasion of EC cells [157]. Similarly, higher SIRT1 expression has been observed in tumors with >50% myometrial invasion and/or lymph node metastasis, suggesting a link between SIRT1 and disease aggressiveness [158]. While SIRT1 upregulation has been generally observed in EC tissues, its expression appears to vary by subtype. Higher levels of SIRT1 have been found in endometrioid EC compared to clear-cell carcinoma [155]. Meanwhile, cytoplasmic SIRT1 expression has been found in all non-endometrioid samples, with no nuclear localization detected [156].

SIRT1 has been shown to play a role in the development of cisplatin resistance and radioresistance in EC. The lncRNA FIRRE has been identified as a prognostic biomarker, and its overexpression has been shown to result in the upregulation of SIRT1 through the sponging of miR-199b-5p, a microRNA that negatively regulates SIRT1 [159]. As a consequence, SIRT1-mediated deacetylation of key autophagy-related protein BECN1 is enhanced, promoting autophagy in EC cells, and leading to autophagy-dependent radioresistance [159].

#### 8.3.2. SIRT2

SIRT2 has been associated with metastatic potential and reduced chemotherapeutic sensitivity and has been proposed as a potential biomarker for EC disease progression and severity. Increased expression of SIRT2 has been observed in EC cell lines. However, no significant difference in SIRT2 mRNA or protein levels between tumor and adjacent healthy endometrial tissues was reported in an analysis of 42 paired patient samples [160]. Despite this, SIRT2 expression was positively correlated with poorer clinical outcomes, including shorter overall survival, reduced progression-free intervals, and higher-risk disease states, supporting its potential use as a prognostic marker [160]. A similar decrease in overall survival was also reported in a separate study, along with a positive association between SIRT2 overexpression and lymph node metastasis, as well as advanced FIGO stage, which reflects disease spread within the female reproductive tract [161]. In functional studies using EC cell lines, SIRT2 overexpression was shown to enhance stemness characteristics, evidenced by increased sphere formation, a greater proportion of CD133^+^ cells, and elevated stem cell frequency, as measured by an ELDA assay [162]. Additionally, SIRT2 overexpression was associated with increased expression of p-MEK1 and p-ERK1/2, leading to activation of the MEK/ERK signaling pathway, which is known to drive metastatic behavior [162]. Co-expressed genes of SIRT2 in tumor tissues were found to be enriched for pathways related to metastasis and EMT, a key process in cancer progression [160].

Like SIRT1, SIRT2 has been implicated in the reduction in chemosensitivity to currently used treatments involving taxanes and platinum analogs. In a cisplatin treatment of SIRT2-overexpressing EC cells, a higher relative cell viability was observed when compared to cells with typical SIRT2 levels [162]. However, no significant change in chemosensitivity was detected when the cells were treated with various concentrations of paclitaxel [162].

#### 8.3.3. SIRT6

SIRT6 has been previously reported to possess anti-EC properties and has been implicated in the mechanism of action of the HMGCR inhibitor fluvastatin in the treatment of EC cells [163]. Treatment with fluvastatin has been shown to suppress the proliferation, invasion, and migration of EC cells while also inducing apoptosis. This suppression is reversed in EC cells in which SIRT6 expression has been silenced [163]. Furthermore, fluvastatin treatment has been shown to increase SIRT6 expression, and the resulting elevation in SIRT6 levels is thought to contribute to its anti-cancer effects [163].

#### 8.3.4. SIRT7

Increased expression of SIRT7 has been observed in human EC tissues, and this upregulation has been positively correlated with EC metastasis and migration [164]. The tumor suppressor PTEN has been identified as a substrate of SIRT7 deacetylase activity, with deacetylation at lysine 260 (K260) linked to its ubiquitination and subsequent degradation by the E3 ligase NEDD4L [164]. A marked decrease in PTEN protein levels has been reported in approximately 45% of poor-prognosis EC cases, potentially associated with elevated SIRT7 expression. Estrogen exposure, a known factor in EC development, has been shown to enhance the interaction between SIRT7 and PTEN [164].

### 8.4. Ovarian Cancer (OC)

OC is the eighth leading cause of cancer-related deaths among women [165]. It is classified into epithelial, germ cell, and stromal tumors, with epithelial tumors being the most malignant. OC is often diagnosed at advanced stages, after metastasis to nearby organs or beyond the abdominal cavity, leading to reduced 5-year survival rates and increased recurrence [166,167,168]. Current treatments include cytoreductive surgery, platinum-based chemotherapy, and targeted therapies such as the anti-angiogenic agent bevacizumab and PARP inhibitors [169].

While the pathogenesis of OC remains poorly understood, risk factors include EM, postmenopausal estrogen therapy, germline *BRCA1/2* mutations, and a family history of OC [168]. The role of sirtuins in OC is complex and often contradictory. Depending on context, they have been reported to function as both oncogenes and tumor suppressors, promoting apoptosis and autophagy in some cases while facilitating tumor growth, angiogenesis, immune evasion, chemoresistance, and metastasis in others [170]. Recent studies exploring these diverse roles are discussed below (Table 6).

#### 8.4.1. SIRT1

SIRT1 is the most extensively studied sirtuin in the context of ovarian health, yet findings remain conflicting. In one study, overexpression of SIRT1 in the tumor microenvironment, mediated by cancer-associated adipocyte-derived extracellular vesicles, upregulated CD24 expression, promoting tumorigenesis and immune evasion in mice [171]. Another study reported that SIRT1 overexpression contributed to paclitaxel resistance in ovarian cancer cells [172]. Pharmacological inhibition of SIRT1 with EX-527 also demonstrated anti-cancer effects, reducing cell proliferation and metastasis [173]. Conversely, treatment of both cisplatin-sensitive and -resistant ovarian cancer cell lines with diallyl trisulfide (Figure 1) inhibited cancer cell proliferation via activation of theSIRT1/AMPK/PGC1α pathway [174]. Additionally, nuclear expression of RXRα and SIRT1 in advanced ovarian cancer was associated with improved overall survival [175].

#### 8.4.2. SIRT2

Similar to SIRT1, SIRT2 is also shown to have both tumorigenic and tumor-suppressive roles, but most of the latest evidence corroborates the former role. A recent study identified SIRT2 as a key pro-metastatic factor in epithelial ovarian cancer [176]. SIRT2 promotes metastasis by deacetylating and stabilizing the transcription factor Slug, a central driver of EMT. SIRT2 was found to shuttle between the cytoplasm and nucleus, where it deacetylates Slug, preventing its acetylation-dependent degradation and enabling it to promote cancer cell migration, invasion, and EMT. High SIRT2 expression correlated with more aggressive disease and poorer patient survival [176]. Interestingly, the tumor suppressor Fn14 was shown to inhibit SIRT2’s pro-metastatic activity by physically interacting with it and blocking its nuclear translocation, thereby facilitating Slug degradation and suppressing metastasis [176]. Pharmacological inhibition of SIRT2 using AGK2 also demonstrated anti-metastatic effects, supporting the Fn14–SIRT2–Slug axis as a novel regulatory pathway in ovarian cancer [176].

#### 8.4.3. SIRT3

A study utilizing ultrasound-targeted microbubble destruction (UTMD) gene delivery demonstrated that SIRT3 functions as a tumor suppressor in ovarian cancer [177]. SIRT3 was found to be expressed at low levels in SKOV3 cells, and its overexpression via UTMD significantly inhibited cell proliferation, migration, invasion, and EMT while promoting apoptosis [177]. Overexpression of SIRT3 reduced the expression of proliferation markers, mesenchymal markers, and anti-apoptotic proteins while increasing levels of epithelial markers and pro-apoptotic factors. Mechanistically, SIRT3 was shown to bind to and downregulate hypoxia-inducible factor-1α (HIF-1α), a key transcription factor involved in angiogenesis and tumor progression. Importantly, UTMD-mediated SIRT3 delivery also effectively suppressed tumor growth in vivo [177].

#### 8.4.4. SIRT4 and SIRT6

A study examining the correlation between sirtuin expression and treatment outcomes found that SIRT4 and SIRT6 had significant prognostic value for both overall survival and progression-free survival, suggesting their potential roles as tumor suppressors in ovarian cancer [178]. However, contrasting evidence from another study showed that SIRT6 overexpression enhanced glycolysis and promoted mitochondrial fragmentation in ovarian cancer cells, processes that facilitate metastasis [179].

**Table 6:** Sitruins in gynecological cancers. Sirtuin inhibitors are highlighted in red, activators are in green, and other agents are in blue.

	Sirtuin Isoform	Expression	Affected Pathway/Mechanism	Pathological Outcomes	Therapeutic Intervention	Ref.
Cervical Cancer	SIRT1	Upregulated	Promotes PD-L1 expression	Immune evasion	EX527	[104]
			Triggers Wnt/β-catenin pathway through MALAT1/miR-124/SIRT1 axis	Tumor growth		[105]
			Suppression of p53 by deacetylation	Tumor proliferation, invasion, and cisplatin resistance	EX527	[110]
			Upregulation of CD38 mediated NAD salvage pathway	Metabolic reprogramming for tumor survival and EMT	NAM	[111]
	SIRT3	Downregulated	Disrupts fatty acid metabolism through inhibition of ACCI	Enhanced tumor growth, poor prognosis		[112]
	SIRT4	Downregulated	Glutamine-metabolism-regulating activity of SIRT4 suppressed by PIKECA mutation	Increased glutamine utilization, proliferation, radioresistance	PIK3 Inhibitor (BYL719)	[113,114]
	SIRT7	Upregulated	SIRT7 positive feedback loop with USP39 and FOXM1	Dysregulation of autophagy and oxidative stress		[115]
			Impaired DNA repair due to SIRT7 phosphorylation by ATM	Chemoresistance and genomic instability		[116]
Breast Cancer	SIRT1	Upregulated	Impaired DNA damage response	Tumor progression		[117]
			Suppression of p53 by deacetylation	Evasion of apoptosis and autophagy	Benzothiazole derivatives, MHY2245	[121,122]
			Increased vimentin expression through SIRT1/AKT axis	EMT, angiogenesis	Montelukast + Sirtinol	[123]
			Amplified ER and GPER signaling in ER+ cells	Oncogenesis, proliferation, chemoresistance	Sirtinol, CHIC 35, and EX 527	[124]
	SIRT2	Upregulated	α-Tubulin hyperacetylation, suppressed STAT3/c-Myc and Snail/MMP pathways	Tumor proliferation	SG3 and Isobavachalcone	[128,129]
			Disrupted post-translational regulation of NAT1 caused increase NAT1 activity	Disrupted acetylation profiles of key proteins	EX527, Sirtinol	[130]
	SIRT3	Downregulated	Mitochondiral dysfunction due to hyperacetylation of MnSOD and NRF2	Chemoresistance to cisplatin and DOX Paclitaxel induced peripheral neuropathy	NR ADTL-SA1215	[136,138,139]
	SIRT5	Downregulated	Reduced glutaminase activity	Increased glutamine utilization, proliferation, chemoresistance	MC3188	[142]
	SIRT6	Upregulated	Repressed transcription of tumor suppressor TBX3 Upregulation of MMP-9 through the MAPK, NF-κB, and AP-1 pathways	Enhanced metastasis		[144,145]
			Unknown	Development of stem-like features	Kaempferol and apigenin	[146]
	SIRT7	Upregulated (early)	SIRT7 is involved in p38-MAPK pathway	May have tumor-suppressive or -promoting role		[148]
		Downregulated (metastatic)	SIRT7 inhibits TGF- β signaling, epigenetically represses TIE2/TEK, and maintains chromosomal stabilty	Predominantly exerts anti-metastatic effects		[148,149,150]
Uterine/Endometrial Cancer	SIRT1	Upregulated	Deacetylation of BECNI to promote autophagy	Higher metastasis Development of chemo and radio-resistance		[158,159]
	SIRT2	Upregulated	Activation of MEK/ERK pathway	Metastasis and EMT		[162]
	SIRT7	Upregulated	Promotes proteolytic degradation of tumor suppressor PTEN by deacetylation	Metastasis and poor prognosis		[164]
Ovarian Cancer	SIRT1	Upregulated	Involved in AMPK/SIRT1/PGC1α pathway Upregulates CD24 expression	May exert tumor-promoting or tumor-suppressing effect	EX-527	[171,173,174]
	SIRT2	Upregulated	Deacetylation and stabilization transcription factor Slug	Cancer cell migration, invasion, and EMT	AGK2	[176]
	SIRT3	Downregulated	SIRT3 downregulates HIF-1α pathway	Angiogenesis, tumor progression		[177]

## 9. Menopause

Aging in women is accompanied by complex physiological changes that increase vulnerability to metabolic and cardiovascular diseases [180,181]. Menopause represents a key milestone in this process, marked by the permanent cessation of the menstrual cycle. The menopausal transition reflects the progressive aging of the female reproductive system and is characterized by a decline in estrogen levels [182]. This estrogen reduction is a central driver of many perimenopausal and postmenopausal health issues including metabolic and cardiovascular disorders, mood disturbances, osteoporosis, osteoarthritis, and cognitive decline [180,181,183,184].

While aging is strongly associated with a decline in NAD^+^ and sirtuin levels across different tissues, decreasing estrogen further exacerbates this effect. Among the seven sirtuins, Sirt1, Sirt3, and Sirt6 have been shown to be downregulated in various tissues of ovariectomized (OVX) rats, a widely used animal model of menopause [185]. Estrogens are known to induce the expression of sirtuins, along with other estrogen-responsive proteins, through activation of G protein-coupled estrogen receptors (GPERs) as well as the classical estrogen receptors ERα and Erβ [185,186,187]. Interestingly, sirtuins also promote the expression and stabilization of estrogen receptors, suggesting the existence of a feedback pathway, although its exact mechanism remains to be elucidated [188,189].

### 9.1. SIRT1

Several animal studies have established strong connections between sirtuin downregulation and the detrimental effects of menopause. Ovariectomy (OVX)-induced non-alcoholic fatty liver disease (NAFLD) has been linked to downregulation of the Erα/Sirt1/PGC-1β axis in liver tissue [186]. Treatment with 17β-estradiol demonstrated a hepatoprotective effect by restoring Erα/Sirt1/PGC-1β signaling [186]. Sirt1 also mediates estradiol-induced Rab9-dependent alternative autophagy through the Sirt1/LKB1/AMPK/Unc-51-like autophagy activating kinase 1 (Ulk1) pathway, which protects the vasculature and delays arterial senescence [190]. Oral supplementation with resveratrol has also been shown to upregulate Sirt1 and ameliorate metabolic and cardiovascular symptoms associated with menopause and hypoestrogenism [191,192,193].

Apart from estrogen replacement therapy and other pharmacological interventions, lifestyle strategies such as calorie restriction and exercise, both aerobic and resistance training (RT), have been shown to significantly improve menopausal symptoms [194,195]. In OVX rats, reduction in Sirt1 in left-ventricular heart tissue led to apoptosis, as evidenced by increased levels of cleaved caspase-3 and Bax, along with decreased Bcl-2 expression [194]. Calorie restriction was able to restore Sirt1 levels and reduce apoptotic markers [194]. High-intensity interval training (HIIT) and RT have also been shown elevate serum Sirt1 levels, with HIIT producing greater benefits in metabolic and cardiovascular parameters and RT being more effective for maintaining and building muscle mass, thereby helping to prevent osteoporosis and osteoarthritis [195].

### 9.2. SIRT3

SIRT3 reduction during menopause contributes to oxidative stress not just in the ovaries but systemically. In vinylcycloxene diepoxide-induced rodent models, reduced Sirt3 levels impaired the deacetylation and activation of superoxide dismutase (SOD), leading to increased oxidative stress in the brain [196]. These rodents displayed depression- and anxiety-like behaviors, which were attenuated by kaempferol treatment through elevation of Sirt3 levels, which enhanced SOD deacetylation [196].

## 10. Osteoporosis (OP)

OP is characterized by a loss of bone mineral density and bone mass, ultimately leading to fragile bones that may fracture or break under minor stresses such as bending, twisting, coughing, or even spontaneously [197]. In the United States, an estimated 10 million people are affected by OP, 80% of whom are women [198]. The disease impacts one in four women over the age of 65. A decline in estrogen during the postmenopausal state is a predominant risk factor, while pre-existing hormonal imbalances or deficiencies, endocrine disorders, and disordered eating further increase susceptibility to bone loss [197,198]. SIRT1, SIRT2, and SIRT7 have been identified as protective against bone loss, with reduced expression observed in OP (Table 7). In contrast, SIRT3 has been reported both as a contributor to increased bone resorption associated with aging and estrogen deficiency [199,200,201] and as a protective regulator that suppresses osteoclastogenesis [202,203].

### 10.1. SIRT1

Vitamin D deficiency can contribute to accelerated bone loss, and reduced SIRT1 expression has been observed under vitamin D-deficient OP conditions [204]. It was reported that vitamin D transcriptionally regulates SIRT1 expression through the vitamin D receptor (VDR). In a vitamin D-deficient mouse model, increased acetylated PGC1α, a transcription factor, was observed as a result of suppression of SIRT1’s deacetylase activity. Both resveratrol treatment and PGC1α knockdown enhanced osteogenesis while reducing senescence and oxidative stress, thereby linking the SIRT1–PGC1α interaction to osteogenesis [204]. In an OVX rat model, treatment with the vitamin D analog Eldecalcitol (ED-71, Table 7) reduced the number of aging cells and ROS while increasing both SIRT1 mRNA expression, SIRT1 activity, and the subsequent upregulation of the downstream Nrf2 pathway via deacetylation of Nrf2 [205]. Similar improvement in SIRT1 expression and activity was seen with Segetalin B (SB, Table 7) treatment, which led to enhanced osteogenic differentiation. This effect was diminished with the addition of a PDL1 inhibitor, linking PDL1 activation to SIRT1 activation [206]. SB treatment also inhibited g-secretase, resulting in downstream inhibition of the Notch1 signaling pathway, revealing the role of SIRT1 in Notch1 signaling, which is activated in OVX mice.

SIRT1 knockdown has been shown to reduce the ability to form new bone in a mouse model [207]. In an OVX mouse model, it was found that stimulation with hydrogen peroxide decreased the levels of SIRT1, HO-1, and Nrf2 [208]. Treatment with ellagic acid (Table 7) restored the expression of SIRT1, HO-1, and nuclear Nrf2 and improved the osteoporotic phenotype, effects that were partially reversed by treatment with a SIRT1 inhibitor [208]. Within an OVX mouse model, treatment with cholesterol sulfate (CS, Table 7) restored bone mineral density, bone volume, and trabecular bone number via the AMPK-SIRT1-NF-κB pathway. CS treatment activated SIRT1 through AMPK and resulted in decreased acetylation levels of known SIRT1 targets, p65K310 and p53K379 [209]. Deacetylation of p65K310 has previously been shown to inhibit NF-kB [210,211]. CS-induced NF-kB inhibition was partially reversed by treatment with an AMPK inhibitor, validating the above mechanism.

It was found that treatment of MC3T3-E1 cells and OVX mice with globular CTRP3 (gCTRP3), a protein with diminished levels in OP, alleviated bone loss and stimulated new bone formation along with increased levels of p-AMPK, SIRT1, and Nrf2. When mice were co-treated with an inhibitor of any of these three proteins, gCTRP3-induced osteoblast differentiation, formation of mineralized calcium nodules, and elevated ALP activity were reversed [212].

Implantation of a novel Icariin/porous magnesium alloy scaffold in a rat model was shown to stimulate the expression of SIRT1 in BMSCs of the rats [213]. This increase in SIRT1 expression was correlated with activation of the Wnt/β-catenin pathway and promotion of osteogenic differentiation, which was responsible for the increased rate of bone repair [213].

### 10.2. SIRT2

In OP, miR-26b is overexpressed in BMSCs and has been shown to interact with SIRT2 [214]. In human chondrocyte C28/I2 cells, an increase in miR-26b expression alongside a decrease in SIRT2 protein levels has been observed. The miR-26b upregulation and SIRT2 downregulation led to the promotion of proliferation and inhibition of apoptosis in osteoporotic chondrocytes. These effects were reversed by silencing SIRT2 in C28/I2 cells [214].

### 10.3. SIRT3

Previously, SIRT3 has been implicated in bone resorption by osteoclasts associated with aging and estrogen deficiency [200,201]. The deletion of SIRT3 in female mice attenuated trabecular and cortical bone loss and suppressed osteoclast-mediated bone resorption, though it did not affect osteoclast numbers [199]. Notably, SIRT3 deletion had no significant impact on bone resorption in male mice [199]. In contrast, another study demonstrated that SIRT3 downregulation in RAW264.7 cells increased RANKL-induced ROS levels [202]. Treatment with Bilobalide (Bil, Table 7) restored SIRT3 expression during osteoclastogenesis, whereas SIRT3 knockdown in the presence of Bil reduced SOD2 levels, elevated ROS production, and activated the NF-kB pathway, ultimately promoting osteoclastogenesis and bone resorption [202]. Similarly, exposure to H_2_O_2_-induced oxidative stress has been shown to reduce SIRT3 levels in MC3T3-E1 cells [203]. Metformin (Met) treatment restored SIRT3 level and attenuated osteoblast apoptosis. These protective effects were abolished with SIRT3 knockdown. In an OVX mouse model, Met treatment reduced trabecular bone loss, thereby mitigating the bone loss characteristic of osteoporosis [203].

### 10.4. SIRT7

BMSC differentiation is reduced in OP [215] and has been shown to be inhibited by both miR-193a and activation of the NF-kB pathway [216,217,218,219]. SIRT7, on the other hand, has been linked to the maintenance of bone growth [220]. In human plasma and bone samples, as well as in an OVX rat model, it has been observed that overexpression of miR-193a was negatively correlated with the downregulation of SIRT7 expression [221]. It was further demonstrated that resveratrol treatment promoted BMSC differentiation by restoring SIRT7 expression through the downregulation of miR-193a. Increased SIRT7 expression inhibited NF-kB pathway via the reduction in phosphorylated p63 and IkBa, two NF-kB pathway proteins [221]. Since NF-κB inhibition has been shown to support bone formation and prevent osteoporotic bone loss [222], these findings underscore the regulatory role of SIRT7.

**Table 7:** Summary of sirtuins in menopause and osteoporosis.

	Sirtuin Isoform	Expression	Affected Pathways or Interaction	Pathological Outcomes	Therapeutic Intervention	Ref.
Menopause	SIRT1	Downregulated	Downregulation of SIRT1/LKB1/AMPK/ULK1 and Erα/SIRT1/PGC-1β signaling pathway	Arterial senescence and NAFLD-induced liver damage	17β-estradiol or resveratrol treatment	[186,190,191,192,193]
			Increased apoptotic markers	Apoptosis	Calorie restriction, HIIT and RT	[194,195]
	SIRT3	Downregulated	Decreased SIRT3-mediated deacetylation of SOD	Increased oxidative stress, depression, and anxiety	Kaempferol treatment	[196]
Osteoporosis	SIRT1	Downregulated	Downregulation of Wnt/β-catenin pathway	Osteogenic senescence	Implantation of Icariin/porous magnesium alloy scaffold	[213]
			PGC1α-SIRT1 interaction	Increased oxidative stress and senescence	Resveratrol treatment	[204]
			Decrease in SIRT1-mediated NRF2 acetylation and AMPK-SIRT1-NF-κB signaling	Reduction in new bone formation and osteogenic senescence	Segetalin B, ED-71, ellagic acid, CS, and gCTRP3 treatments	[205,206,207,208,209,210,211,212]
			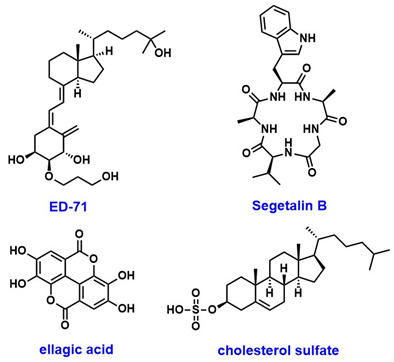			
	SIRT2	Downregulated	Upregulation of miR-26	Promotion of proliferation and inhibition of apoptosis		[214]
	SIRT3	Downregulated	Increased RANKL-induced ROS levels and activated NF-kB pathway	Bone resorption and osteoclastogenesis	Bilobalide and metformin treatment 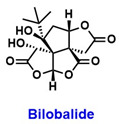	[202,203]
	SIRT7	Downregulated	Upregulation of NF-kB pathway	BMSC senescence	Bone loss	[216,217,218,219,220,221]

## 11. Conclusions and Perspectives

Accumulating evidence highlights sirtuins as central regulators of pathophysiological processes across a broad spectrum of women’s health. From reproductive disorders such as PCOS and endometriosis to pregnancy complications, gynecologic oncogenesis, and the metabolic shifts of menopause, sirtuins integrate metabolic, oxidative, and inflammatory signals to modulate cellular responses. The therapeutic potential of sirtuin modulation is compelling. Enhancing sirtuin activity, either through NAD^+^ boosters such as NMN and NR or direct activators (such as SRT2104 for SIRT1), holds promise for conditions with few effective treatments. For example, SIRT1 activation improves insulin insensitivity in GDM [83], alleviates symptoms in PE [66], and promotes bone formation in OP [209]. The paradoxical function of sirtuins in cancer, as both tumor suppressors and promoters, emphasizes the importance of isoform-specific and context-dependent targeting for future precision medicine.

Despite the varied disease contexts, the recurring connections between certain biochemical pathways and specific sirtuin isoforms are notable and summarized below. SIRT1, due to its nuclear localization, is involved in suppression of nuclear factors like NF-κβ and p53. In addition, the SIRT1-AMPK axis is a key regulatory pathway involved in energy metabolism. Both these functions position SIRT1 generally as a driver of cell growth and metabolic flexibility and suppressor of inflammation. Similarly, SIRT2 is strongly associated with glucose metabolism and regulation of autophagy, whereas SIRT3 is associated with activation of ROS-scavenging proteins and maintaining mitochondrial health. Generally, these functions of SIRT1, SIRT2, and SIRT3 need to be activated in PCOS, GDM, fertility issues, and menopause, while they need to inhibited in cancers and endometriosis where aberrant cell growth is the problem. SIRT4-SIRT6 have more variable roles in disease pathogenesis, especially cancers. However, SIRT4 is primarily associated with regulation of glutamine metabolism and SIRT6 with transcriptional regulation of various proteins through histone deacetylation. These unifying patterns across diseases indicate clear delineation of tasks among the different isoforms and also reflect the potential for versatile use of pharmacological modulators of sirtuins.

While sirtuins and NAD^+^ are popularly associated with longevity, they become more crucial in women. Due to dramatic estrogen decline during menopause, women experience an accelerated decline in sirtuins and NAD^+^, resulting in faster aging of tissues and highly dysregulated metabolism. These effects make women vulnerable to several age-related diseases as early as in their 40s, in contrast to men, where aging is slower and more gradual. Therefore, NAD boosters and sirtuin activators can exert more protective effects in and have potential to be used with or without hormone replacement therapies.

The research on sirtuins in women’s health has advanced significantly during the last few decades, moving from recognizing their association to beginning to understand the underlying mechanisms. Despite all the challenges, selective sirtuin modulators hold immense potential to shift the therapeutic paradigms. By directing efforts in isoform specificity, context-dependent applications, and clinical validations, innovative and effective therapies targeting human sirtuins can be delivered to extend the lifespan and health span of women.

## Data Availability

No new data were created or analyzed in this study.
